# Exploring the relationship between female language teachers' agency and teaching innovation in the PRC

**DOI:** 10.3389/fpsyg.2023.1032943

**Published:** 2023-02-23

**Authors:** Xi Chen, Xinmin Zheng

**Affiliations:** ^1^School of Chinese Studies and Exchange, Shanghai International Studies University, Shanghai, China; ^2^School of Education, Shanghai International Studies University, Shanghai, China

**Keywords:** teacher agency, teacher professional development, teaching innovation, female English teachers, changing curricular landscape

## Introduction

Over the last decade, teacher agency has been increasingly studied (Biesta and Tedder, 2007; Ollerhead, 2010; Priestley et al., 2012, 2015; Oolbekkink-Marchand et al., 2017; Tao and Gao, 2017; Bergh and Wahlström, 2018; Jenkins, 2020; Martin, 2020; Ashton, 2021; Huang, 2021; Huang and Yip, 2021). Those results have revealed that teacher agency is an influential factor for teacher professional learning, teaching innovation, school improvement and sustainable educational change (Deters, 2011; Feryok, 2012; Toom et al., 2015; Tao and Gao, 2017; Miller and Gkonou, 2018; Weng et al., 2019; Huang, 2021; Ashton, 2022; White et al., 2022). More studies (see Ng and Boucher-Yip, 2016; Kayi-Aydar, 2019) have been conducted to examine either how researchers adopt relevant theoretical frameworks and research methods in quest of understanding language teacher agency, or explore language teacher agency across interdisciplinary perspectives, for instance, to understand how teachers respond to top-down English language planning and policy decisions.

In a systematic literature review, Ngo (2021) identifies six major themes about recent teacher agency research studies, which include (1) teacher agency enactment, (2) the role of teacher cognition, (3) influential factors, (4) implementing professional development interventions, (5) teacher agency outcomes and (6) teacher agency change trajectory. Besides, some research studies (for example, Gratacós et al., 2021) focus on teachers' personal capacities of their self-efficacy. It is apparent that self-efficacy was identified relevant for teachers to respond to the challenges of their settings, implementation strategies, and contextual factors that can affect student performance. As a result, it is obvious that teacher agency can be extrapolated in terms of its source, enactment and outcomes. Teachers' capacities, implementation strategies, and contextual factors jointly determine the achievement of their professional agency (Mei Kin and Abdull Kareem, 2021).

The growing body of literature on language teacher agency provides us with a better understanding of how teachers enact agency, together with necessary theoretical and analytical approaches for supporting researchers how to conduct teacher agency research. According to Hitlin and Elder (2007), when routines break down, teachers are required to reflect on their practice and to innovate or make changes (see Ruohotie-Lyhty, 2013; Ruohotie-Lyhty and Moate, 2015; Leijen et al., 2020; Ehren et al., 2021). Thus, we hold that there is still much to learn about the complex way in which how individual teachers exercise their agency in their day-to-day teaching practice with the changing environments in different contexts, female teachers' agency in particular.

Building on the previous research work, focusing on examining the tertiary female English teachers, the monograph *Understanding the Professional Agency of Female Language Teachers in a Chinese University: Rhetoric and Reality* published by Routledge (Ruan, 2021) is an intelligible, illustrative and illuminating book. The research study focuses on describing how the case study female English teachers intended to fulfill their aspirations by actualizing their agency competence, through a series of goal setting, envisioning, acting and reflecting practices so as to make decisions and finally achieve their goals. The research study also demonstrates that female language teachers had to work even harder to establish themselves. On the whole, the research study offers insightful suggestions to language education in both China and broader areas globally. We believe the research book will appeal to researchers studying teacher education and foreign (English) language teaching, university teachers, especially female foreign language teachers, PhD students and graduate students, as well as career women. Hence, we have no hesitation in recommending it to anyone that is interested in the interplay of teacher agency and teacher professional development.

## The study

The monograph on *Understanding the Professional Agency of Female Language Teachers in a Chinese University* (Ruan, 2021) consists of eight chapters, which covers a range of theoretical, thematic, and analytic issues, by providing profound examples, solid data, instructive questions, logical arguments and useful recommended references.

Chapter 1 expounds on the research background, the author's personal research motivation, and key issues of research planning. The background information of the first chapter describes the history and nature of teacher professional agency research problems with reference to the existing worldwide literature and then firmly locates its research in the largest language educational system in the world, that of the People's Republic of China (PRC). Besides, the chapter clearly indicates the root of the problem being studied, appropriate context of the problem in relation to theory, research, and/or practice, its scope, and the extent to which previous studies have successfully investigated the problem, noting, in particular, where the research gap exists and the significance of this research study.

Chapter 2, by reviewing and synthesizing the relevant research studies on teacher agency, highlights the significance of teachers' beliefs and teacher' practices in the first place, so as to help readers establish a general understanding of the relationship between teachers' beliefs and realization of teacher development. Then the chapter sets out to reconceptualize teacher agency by redefining it as teacher agency is not merely a personal attribute, but includes pedagogical efforts made by teachers and proactive endeavors in teaching engagement and teacher learning activities, which widens and deepens the examining dimensions of the previous studies by adding a number of new factors, i.e., self-efficacy, self-regulation, persistence and sense of moral responsibility. After that, the chapter narrows down the reviewing on language teacher agency, gender identity, work-life balance and female teacher agency in particular and finally the chapter raises cohesive and logical research questions to explore the dynamic entity of teacher agency at a selected institution in PRC.

Chapter 3 demonstrates how the author reflects on her methodological considerations of the research study. As this study intends to reveal female language teachers' agency, the author finds it necessary to clarify the reason why she adopted qualitative method to conduct the study, furthermore, the author also identifies a series of methodological issues, such as narrative questionnaire, metaphor, timeline, interview, classroom observation, data, collection, data presentation, type of analysis, and level of analysis adopted throughout the following chapters. Those philosophical and methodological considerations set an important tone for thick-describing the dynamic entity manifested in the ongoing negotiation of agency belief, agency practice, and agency inclination, as well as the interaction of individual and the environment as the research questions guide.

Chapters 4, 5, and 6 describe in detail how the three female teachers' agency beliefs, agency competence, and agency inclination negotiate the relevant perspectives in their contextual workplaces, such as language policies, curriculum, institutional culture, collegial network, university administration, students' learning motivations, research requirement and other pressure and anxiety. With different agency beliefs, agency competence and agency inclination, on the one hand, when facing many challenges, the three case study teachers had to rely on their learning experiences to survive their early teaching responsibilities, on the other hand, as times went on, they must also seek possibilities in change to respond to the interminable contextual requests and adapt to the new routines. Each chapter gives a vivid account of how each teacher mediated diverse conflicting situational factors in their specific context, including the diverse needs of students, collegial cooperation, institutional reform, university administration, and personal professional development, which provides a solid data source for the author to examine and explore the dynamic entities of the attributes and natures of the female teachers' professional agency in the discussion chapter.

Chapter 7 discusses the issues that arise from a comparison of the three case study teachers. It sets out to illustrate the three female teachers' professional agency by demonstrating the salient features of their agency beliefs, agency competence, and agency inclination, as well as the independent relations within the three aspects. Then the chapter focuses on analyzing and examining the factors affecting the three case study teachers' teaching and research behaviors. The purpose of doing so is to propose a model for teacher professional innovation and change in the Chinese context by illustrating the impact of the external, internal and situated forces on teachers and on the resulting professional agency change. From the discussion, it argues that female language teachers in China are open to new teaching professional landscape; however, the process of their agency change is not radical, but incremental and pragmatic, constrained by internal, external and situated forces.

Chapter 8, the concluding chapter, summarizes the findings of the study, synthesizes the study's theoretical and empirical contributions and points out its limitations and implications for teacher agency professional development in China before it puts forward suggestions for future research.

## Discussion

Female language teachers constitute the larger population as compared to their male counterpart (MacNell et al., 2015; Alieto et al., 2020). It is obvious that they play an essential role in the success of the implementation of the latest language-in-education policy as well as teacher learning (Ko, 2013). One of the most significant strengths of this monograph lies in its focus on exploring female English teacher agency in PRC, which has not yet been extensively and systematically studied. The aim of this study is appealing as it expands a better understanding of female English teacher agency in terms of the relationship between teachers' agentic conceptions and their agentic behaviors and thus fills the void of the previous research gap. What is more, with comprehensive and up-to-date overviews and analysis, the study has constructed an important theoretical framework. It provides researchers with an accessible guidance on how to probe into trichotomy of agency beliefs, agency competence and agency inclination, as well as their interplay positioned in the socio-cultural context so that the researchers can put the theoretical and methodological issues of teacher agency analysis into their own practice.

Nonetheless, we think focusing only on the institutional and micro aspects of teacher agency could be too limiting as teacher agency, similarly to teacher identity, is not a constant or immutable entity one possesses; instead, it is also much influenced by macro level, for instance, educational policy (Biesta et al., 2015). It is clear that teachers are social beings, and they are also involved in the process of learning and passing on ideas, beliefs, and practices that are important to them. This is how teachers express their identities and commitments and sustain their worldviews or ideologies. According to Freeden (2013), ideological approach has made significant methodological, theoretical and empirical advances, in disciplines as diverse as political theory (Freeden, 1996), intellectual history (Skinner, 2002), political psychology (Jost et al., 2009), discourse analysis (Chouliaraki and Fairclough, 2010), political science (Carmines and D'Amico, 2015), sociology (Boudon, 1989), and socio-cultural studies (Shelby, 2003). In order to better capture the complexities of teacher agency and institution reforms in a more comprehensive and in-depth way, we suggest approaches such as the ideological perspective should be incorporated into Ruan's theoretical framework. The reason is that the study of female teacher agency is a field where considerable controversy continues to exist and an opportunity for cordial debate could help resolve differences in the field.

Drawing on a longitudinal project, this monograph focuses on the micro-aspects of emergent teacher agency of the three female English teachers at a School of English in a Chinese university with a focus on the causative social factors that mediate this construct. The data of this monograph is very rich, which includes the three female teachers' interviews, stories, teaching practice both online and offline and interactions with their colleagues and others. Evidently, as those factors are not isolated but intertwined, and therefore to better enable the study to conduct an in-depth exploration of such an intricate phenomenon within the focused context, we believe it is feasible to employ the qualitative case study methodology (Njie and Asimiran, 2014).

It is acknowledged that in education research, qualitative and quantitative researchers employ different methods to achieve different goals (Yazan, 2015). It is not meaningful to argue or justify which is necessarily more superior to another. Instead, more and more researchers tend to adopt an integrated or mixed-method approach to education research inquiry (Trafimow, 2014). From our point of view, researchers should consider carefully how such integration might occur by adopting a continuum instead of a dichotomy of generalizability. To be more specific, we hold that researchers should consider how that continuum can be related to the types of research questions raised as the nature of research questions of a research study would determine the selection of a research approach.

As far as we are concerned, despite all the strengths of the monograph described above, readers may find the research study more comprehensive and illuminating if quantitative methods of data collection and analysis were incorporated in the study. This is because qualitative research alone is often insufficient to make population-level summaries (Mason, 2002). In reality, policy makers may not value the descriptive position and therefore may not recognize the importance of a qualitative study and alike. However, it should be noted that many qualitative multiple-case studies have proved appropriate, feasible and fruitful in employing only qualitative data (e.g., Huang et al., 2019, 2021; Chen and Huang, 2022; Ma, 2022).

Finally, as the gender issue is one of the most prominent factors of this study, to some extent, we can see the study did make effort to collect data relating to the feminine roles and also made analysis of the data to be more meaningful. However, it was a pity that the study did not make a clear description to tell how it set up a feasible protocol to systematically collect the data and analyze the data. The fact that the permeability between family and work scopes produces work-family conflict is well-established in literature (Cerrato and Cifre, 2018). It has always been difficult for female teachers to balance a thriving career and a happy family life. This is because being a full-time working teacher comes with sessions of stress and guilt for not being able to give sufficient time to family and work. If the study had paid closer attention to this aspect and had dug the collected data deeper, the research findings would be more convincing and the interpretation would be more inclusive.

## Conclusion

To summarize, this monograph adopts a socio-cultural approach to examine three female English teachers' professional agency in PRC by exploring their different stages of changes, which has significantly extended our understanding of the relationship between teacher agency and teaching innovation. The research study explores the scenario carefully and profoundly to describe and interpret how the case study teachers enacted their professional agency by partaking in multifarious teaching commitments, academic research, and teacher learning activities, which is a dilemma for many young female English teachers and a predicament for tertiary effective professional development to scaffold and support. This monograph is lucid, objective, solid and logical. Indeed, the monograph has depicted an interesting dynamic entity in which how female English teachers struggled and reshaped themselves and even tried to balance amid their self-discrepancies in a changing curricular landscape and their agentic experiences in narrowing the existing gulfs between their idealized visions. Thus, the monograph has provided us with a clear-cut and valuable theoretical framework to further examine English teacher agency in Chinese context of 21st century as well as in the rest of the world.

## Author contributions

XC and XZ selected the monograph together and co-wrote the opinion article. As XC's supervisor, XZ provided XC with insights and suggestions during the writing and polished and finalized the article. All authors contributed to the article and approved the submitted version.
